# The influence of articulatory suppression on the control of implicit sequence knowledge

**DOI:** 10.3389/fnhum.2012.00208

**Published:** 2012-07-13

**Authors:** Vinciane Gaillard, Arnaud Destrebecqz, Axel Cleeremans

**Affiliations:** Consciousness, Cognition and Computation Group, Université Libre de BruxellesBrussels, Belgium

**Keywords:** inner speech, articulatory suppression, sequence learning, control

## Abstract

The present study investigated the consciousness-control relationship by suppressing the possibility to exert executive control on incidentally acquired knowledge. Participants first learned a sequence of locations through a serial reaction time (SRT) task. Next, to assess the extent to which the incidentally acquired knowledge was available to executive control, they were asked both to generate the learned sequence under inclusion instructions, and then to avoid the generation of the learned sequence under exclusion instructions. We manipulated the possibility for participants to recruit control processes in the generation task in three different conditions. In addition to a control condition, participants generated sequences under inclusion and exclusion concurrently with either articulatory suppression or foot tapping. In a final recognition task, participants reacted to old vs. new short sequences (triplets), and judged, for each sequence, whether it had been presented before or not. Results suggest that articulatory suppression specifically impairs exclusion performance by interfering with inner speech. Because participants were nevertheless able to successfully recognize fragments of the training sequence in the recognition task, this is indicative of a dissociation between control and recognition memory. In other words, this study suggests that executive control and consciousness might not be associated in all circumstances.

Executive control and consciousness are typically assumed to be associated (see Moors and De Houwer, 2006; Hommel, 2007 for reviews): one can only control the knowledge that one is aware of. Dehaene and colleagues “neural workspace” hypothesis, for instance, explicitly rules out the possibility that an unconscious process can modulate high-level, conscious processes (Dehaene and Changeux, 2004). However, evidence that executive control is possible in the absence of awareness has recently been obtained with masked priming (Heinemann et al., 2009; van Gaal et al., 2009; Kiefer and Martens, 2010; Capa et al., 2011).

Using a different method, the present study aimed at assessing whether knowledge that one cannot control nevertheless remains available to awareness. Participants first learned a sequence of locations through a serial reaction time (SRT) task (Nissen and Bullemer, 1987). Next, to assess the extent to which the incidentally acquired knowledge was available to executive control, they were asked both to generate the learned sequence under inclusion instructions, and then to avoid the generation of the learned sequence under exclusion instructions. Comparing the knowledge produced when people are directly instructed to recall it and when they are directly instructed to avoid its recall makes it possible to assess the extent to which people are able to control the influence of the acquired knowledge (Destrebecqz and Cleeremans, 2001; Wilkinson and Shanks, 2004). Finally a direct recognition task assessed awareness of the material. Crucially, people's ability to control the expression of the learned knowledge was manipulated by asking participants to perform a secondary task during generation. Thus, in addition to a control condition, participants generated sequences under inclusion and exclusion instructions concurrently with either articulatory suppression or foot tapping. The rationale behind this manipulation stems from the well-established relationship between language, inner speech, and cognitive control. Cragg and Nation (2010) recently surveyed the studies showing a parallel development of language abilities and cognitive control. Indeed, after the seminal “Thought and language” book by Vygotsky (1962), there is no doubt that language plays a role in guiding children's own thinking and behavior. However, the exact implication of inner speech in cognitive control continues to be debated. Cragg and Nation (2010) suggest that language may be implicated in selecting and activating the relevant task set, in keeping track of the task (or item) order, and in retrieving the relevant task goal, especially when conflicting information is present. In the following, we briefly review evidence that suppressing inner speech is indeed detrimental to controlled processes.

## Suppressing inner speech as a means of interfering with executive control

One of the foremost methods to study the role of inner speech in higher-level cognition consists of “relatively simple articulatory interference procedures” (de Guerrero, 2005, p. 108). For example, in seeking to induce perseverative errors in healthy participants and compare their performance with frontal patients, Dunbar and Sussman (1995) administered the Wisconsin Card Sorting Test (WCST) concurrently with either an articulatory suppression or a tone detection secondary task. They observed more perseverative errors when a category is changed for the first time, in the articulatory suppression condition only. In other words, perseverative errors can be induced in the WCST by blocking inner speech, because participants loose track of the task rules.

Concurrent articulatory suppression has also been extensively used to hinder task preparation in the task-switching paradigm. In such studies, participants complete or verify lists of addition and subtraction problems, or perform parity, magnitude, letter, color, or shape judgments in blocked vs. alternating form (Baddeley et al., 2001; Emerson and Miyake, 2003; Miyake et al., 2004; Saeki and Saito, 2004, 2009; Bryck and Mayr, 2005; Saeki et al., 2006). Concurrently, they have to perform an articulatory suppression task, which may consist in reciting the days of the week or the months of the year (Baddeley et al., 2001), in saying “the” or “da” repeatedly (Baddeley et al., 2001; Saeki and Saito, 2004, 2009; Bryck and Mayr, 2005; Saeki et al., 2006), in saying the sequence “a–b–c” repeatedly (Emerson and Miyake, 2003) or in repeating “Tuesday” or “Thursday” (Miyake et al., 2004). Articulatory suppression is typically contrasted with a non-verbal secondary task, such as foot tapping, and with a control condition without any secondary task at all. Overall, data indicate that disrupting inner speech via articulatory suppression specifically impairs task-switching performance, above and beyond the detrimental effects imposed by the requirement to perform a dual task.

Articulatory suppression also makes it difficult to override prepotent responses. For instance, blocking inner speech (“inner voice”) increases impulsive responding in a Go/No-Go task (Tullett and Inzlicht, 2010). Participants make more “Go” responses when they say the word “computer” repeatedly than when they continuously draw circles with their non-dominant hand. This is even more so in a switching version of the Go/No-Go task, which requires more self-control. As a consequence, Tullett and Inzlicht (2010) insist on the specific role that verbal resources play in self-control.

It is worth noting that research related to the interplay between language development and the development of action control also points in the same direction. More specifically, Karbach et al. (2011) demonstrated the positive influence of verbal relevant self-instructions for action-effect learning in 4-year-old children. In the same vein, Kray et al. (2008) provided evidence that the deficits in task-switching ability usually observed in younger children and older adults can be counteracted by verbal labelling. The action control benefits associated with verbalization appear to follow a U-shaped developmental trend across the lifespan.

Importantly, regardless of the apparent procedural differences and the specific goals of each study, the general conclusion arising from all these data is that disturbing inner speech has detrimental effects on executive control processes. In this light, inner speech thus serves as an internal self-cuing device that is particularly helpful when endogenous control is required. In other words, inner speech helps drive action in complex situations where information from the immediate past is needed. Building on this conclusion, the present study addresses the question of the relationship between consciousness and control by suppressing the possibility of exerting executive control on knowledge in implicit sequence learning.

Implicit sequence learning is the ability to learn sequential regularities without intending to learn (see Perruchet, 2008; Shanks, 2010). In a typical SRT task (Nissen and Bullemer, 1987), a visual target moves from location to location following a fixed sequence. The task is presented as a speed test in which participants have to track the target by pressing the corresponding keys as fast as possible. The absence of instructions regarding the existence of an underlying sequence makes it unlikely that participants develop any intention to learn its regularities. Nonetheless, they typically show increasing sensitivity to the sequential regularities contained in the sequence as training progresses, as demonstrated by gradually faster responses to predictable locations vs. novel locations (as when the training sequence is suddenly replaced by another). Under these circumstances, participants typically exhibit limited awareness of the sequential regularities (Cleeremans et al., 1998), and sequence learning therefore constitutes an excellent example of implicit learning. Here, at the end of the SRT task, people performed two forced-choice tasks, as in Destrebecqz and Cleeremans (2001). Participants first performed a generation task in which they had to freely generate a sequence under inclusion and exclusion instructions. In a final recognition task, participants were asked to react to old vs. new short sequences (triplets), and to decide, for each sequence, whether it had been presented before or not.

## Aims and hypothesis of the present study

The generation task is of particular interest here because it requires executive control. The specific version of the generation task we used here is based on Destrebecqz and Cleeremans (2001) and includes two phases. Participants first perform the generation task under inclusion instructions. They are asked to generate a sequence that resembles the training sequence as much as possible. Next, they perform the generation task under exclusion instructions, that is, they are asked to avoid reproducing the training sequence (that is, to generate a sequence that is as different as possible from the trained sequence). According to the Process Dissociation Procedure (Jacoby, 1991), generation under inclusion instructions constitute a facilitation task because both explicit and implicit knowledge may help participants generate the training sequence. In contrast, generation under exclusion instructions is an interference task because explicit and implicit knowledge of the repetitive pattern act in opposition: Only conscious, controlled knowledge can help participants avoid producing the training pattern. Thus, observing that fragments of the trained sequence are generated under exclusion instructions can only reflect lack of control.

Based on existing evidence that inner speech supports executive control processes, we assumed that it plays a specific self-cuing role in the generation task. Specifically, we assumed (based on informal interviews) that most participants verbally recode the material and use such verbal codes to organize their memory of the sequence and drive their generation responses, particularly under the difficult exclusion instructions. Thus, if this assumption is correct and that inner speech is indeed involved during generation, then blocking inner speech (through a concurrent articulatory suppression task) should make it particularly difficult for participants to override the tendency to generate training triplets under exclusion instructions. Articulatory suppression should not have such a strong influence under inclusion instructions, however, because generating the trained sequence can also be supported by implicit, automatic processes that do not depend so much on inner speech.

To sum up, the present study aims at disrupting executive control processes in the generation phase of an incidental sequence learning task. All participants were exposed in the same manner to the training sequence during the SRT task, and should thus all have learned the sequential regularities of the material to the same extent. They then performed the generation task (under both inclusion and exclusion instructions) in three different conditions: with concurrent articulatory suppression, with foot tapping, or without any secondary task. We predicted that articulatory suppression would specifically impair exclusion performance, in that participants should generate more elements from the training sequence in this condition, as compared to the control and foot tapping conditions. Finally, since all participants underwent the same recognition task as the final test of the study, we expected them to perform similarly in being able to differentiate between old and new sequential fragments. Observing a dissociation between generation and recognition performance in the articulatory suppression group would suggest that participants had acquired at least some conscious sequential knowledge but that they were nevertheless unable to exert control on it under exclusion instructions.

## Materials and methods

### Participants

Fifty-seven students aged 18–26 years from the Université Libre de Bruxelles received course-credits for taking part in the experiment. They were all unfamiliar with the SRT task. Nineteen of them were randomly assigned to each of three different conditions: articulatory suppression, foot tapping, and control. All participants had normal or corrected sight.

### Procedure and material

The experiment consisted in an SRT task, followed by the generation and recognition tasks. The SRT and recognition tasks were identical in all conditions, whereas the generation task differed according to the experimental conditions (articulatory suppression, foot tapping, and control). The display consisted of four dots arranged horizontally on the computer screen and separated by intervals of 3 cm. The stimulus was a small black circle 0.4 cm in diameter that appeared on a white background, centered 0.4 cm below one of the four dots. Each screen position corresponded to a key on the computer keyboard. The spatial configuration of the keys was fully compatible with the screen positions.

Participants performed a serial four-choice reaction time task during 15 training blocks of 96 trials, for a total of 1440 trials. On each trial, a stimulus appeared at one of the four possible screen locations. Participants were instructed to respond as fast and as accurately as possible by pressing on the corresponding key with the index and middle finger of each hand. Each block of trials began at a different point in the sequence. The target was removed as soon as a key had been pressed, and the next stimulus appeared after a 250 ms interval (i.e., RSI = 250 ms). Erroneous responses were signaled by means of a tone. Short rest breaks occurred between any two experimental blocks. Two second order conditional sequences (SOC1 = 342312143241 and SOC2 = 341243142132) were used in the SRT task. In each group, half of the subjects were trained on SOC1 during the first 13 blocks and during block 15; and on SOC2 during block 14 (that is, the transfer block). In this case, we will consider SOC1 as “own” sequence and SOC2 as “other” sequence. This design was reversed for the other half of the subjects. At the end of the SRT task, participants were informed that the dots had followed a repeating pattern. The two direct tests were then administered.

First, participants performed a generation task under inclusion and exclusion instructions (Destrebecqz and Cleeremans, 2001). A single stimulus appeared in a random location. Participants under inclusion instructions were required to generate a sequence that resembled the training sequence as much as possible. Subsequently, participants under exclusion instructions were required to generate another sequence (i.e., to try to avoid reproducing the sequential regularities of the training sequences). In both generation tasks, participants were also told not to repeat responses. The stimulus moved whenever participants had pressed one of the keys, and appeared at the corresponding location after a 250 ms RSI. In all conditions, a metronome was set to beat at the rate of 80 beats per min (i.e., one beat every 750 ms). Groups differed during the generation task: two groups of participants performed the task with a concurrent articulatory suppression or foot tapping secondary task, whereas the control group performed the generation task alone. The experimenter first described the secondary task and demonstrated how to perform it. Participants were instructed to say “ba-ba-ba” repeatedly once per metronome beat in the articulatory suppression condition, or to tap their dominant foot once per beat in the foot tapping condition. After receiving these instructions and watching the demonstration, participants practiced the secondary task to ensure that the task requirements were clear and that they could perform the task correctly. Then they performed the generation task (both under inclusion and exclusion instructions) in combination with the appropriate secondary task. Performance in the foot tapping and articulatory suppression tasks was closely monitored by the experimenter, who reminded the participants to keep up with the metronome when necessary. The procedure for the control condition was the same as that used for the two secondary task conditions, except that there was no secondary task to perform concurrently with the generation task. The metronome was nevertheless operating in this condition so as to equate the level of external noise to that in the two dual-task conditions. Generation scores were computed as the number of “own”, “other,” and “neither” triplets generated under inclusion and exclusion instructions separately. An “own” triplet is a triplet that was part of the training sequence; an “other” triplet is a triplet that was part of the transfer sequence; a “neither” triplet is a triplet that was neither “own” nor “other”. The maximum number of “own” or “other” triplets was 96.

Finally, participants performed a triplets recognition task, as in Shanks and Johnstone (1999). Participants reacted to 24 fragments of three trials. Twelve were part of SOC1 and 12 were part of SOC2. Participants were asked to respond to stimuli as in the SRT task, and then to provide a rating of how confident they were that the fragment was part of the training sequence. Ratings involved a six points scale where 1 = “I am certain that this fragment was part of the training sequence” and 6 = “I am certain that this fragment was not part of the training sequence”. It was emphasized to participants that they had to respond as fast as possible to the stimuli. Both ratings and reaction times were recorded.

## Results

Prior to each analysis of variance (ANOVA), data were tested with Mauchly's test of sphericity. Where sphericity was of concern, the degrees of freedom were modified with the Greenhouse-Geisser epsilon and effects are reported significant according to the adjusted alpha level. The data from one participant in the articulatory suppression condition was discarded because he did not follow the instructions in the generation task.

### Serial reaction time task

Participants trained with SOC1 and SOC2 were combined in all analyses. RT analyses were conducted for correct responses across 15 blocks. RTs associated with the first two stimuli of each block were excluded, because their locations could not be predicted. Mean error rate was very low (less than 5% of the trials) and did not vary between conditions (*F* < 1, *p* > 0.5). Errors are not discussed further.

**Figure 1:**
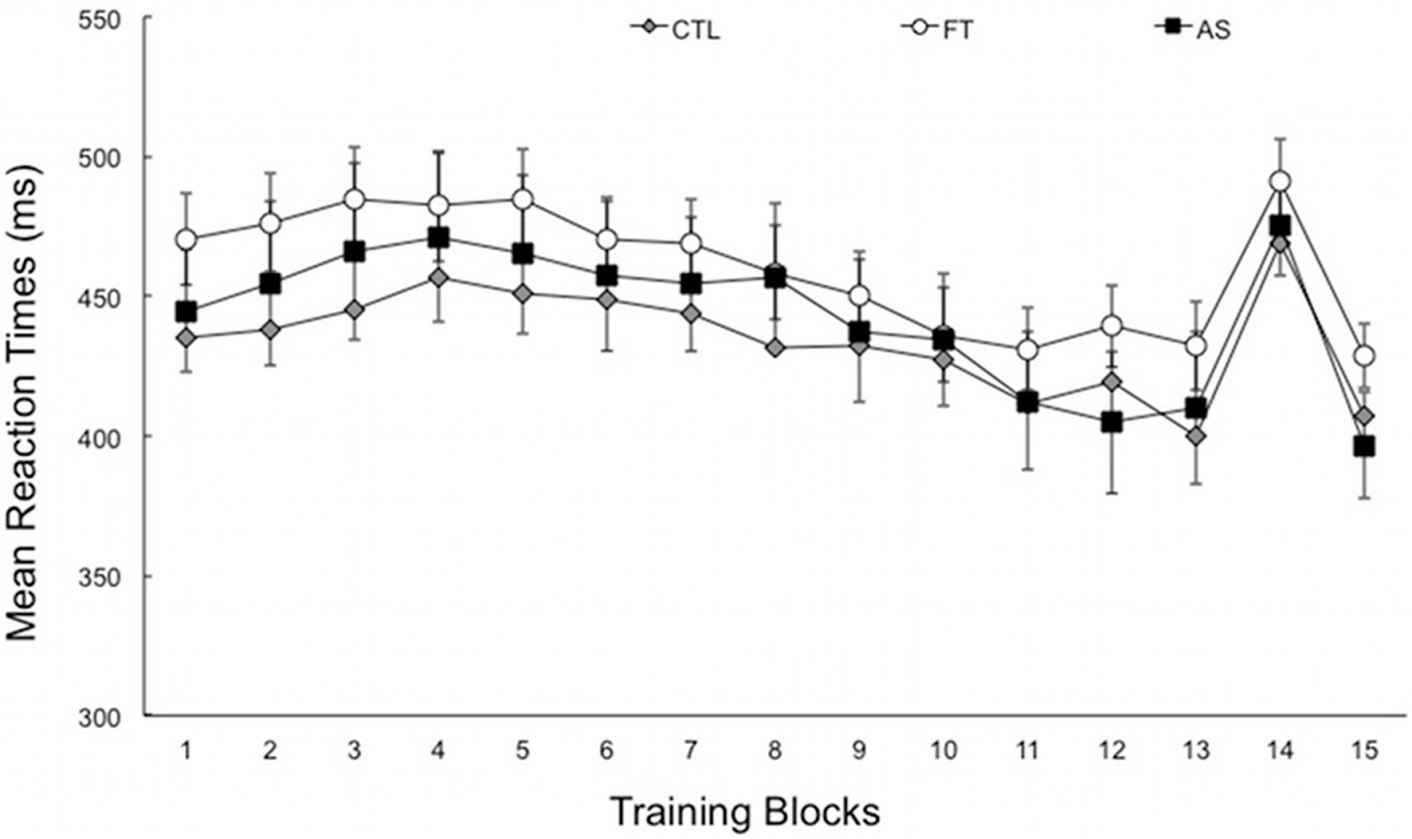
**Mean reaction times for each training block, plotted separately for the three conditions: articulatory suppression (AS), foot tapping (FT), and control (CTL) groups.** Block 14 corresponds to the transfer block. Recall that the experimental setting did not differ during SRT task (data are plotted separately for clarity). Error bars represent standard errors.

Figure 1 shows the average RTs obtained over the entire experiment, plotted separately for the three generation conditions (as a reminder, all participants performed the SRT task under the exact same conditions). A first ANOVA with blocks 1–13 as a within-subjects variable and condition (articulatory suppression, foot tapping, and control) as a between-subjects variable only revealed a significant effect of block, [*F*_(4.83, 256.22)_ = 15.99, *p* < 0.001, η^2^_*p*_ = 0.23]. There was no effect of condition, and no blocks × condition interaction (*Fs* < 1, *ps* > 0.5). As can be seen on Figure 1, this suggests that the overall RTs decrease with practice—from 461.25 ms (*SD* = 88.67) in the first six blocks down to 432.99 ms (*SD* = 78.61) in the last seven blocks. As expected, this decrease does not differ between groups. More importantly, the transfer effect, as induced by the presentation of a different sequence on block 14, gives an indirect index of sequence learning. An ANOVA with transfer (block 14 vs. the mean of blocks 13 and 15) as within-subjects variable and condition (articulatory suppression, foot tapping, and control) as between-subjects variable yielded a significant effect of transfer, [*F*_(1, 53)_ = 151.97, *p* < 0.001, η^2^_*p*_ = 0.74]. Overall, reaction times increased by 65.86 ms (*SD* = 5.29) when the training (own) sequence was changed to another sequence in block 14. As in the previous analysis and as expected, there was no effect of condition, and no transfer × condition interaction (*Fs* < 1, *ps* > 0.4).

This suggests (1) that RTs increased significantly in the three conditions when the sequence was modified and (2) that this RTs increase was of same extent in all three conditions, suggesting equivalent levels of sequence learning. Taken together, SRT task results show that sequence learning was observed in the three groups of participants. Indeed, the RTs decreased with practice when the same sequence is presented to the participants, increased when the sequence was modified and decreased again when the training sequence was put anew. We now examine whether participants differ in their ability to project their knowledge of the sequence in generation and recognition tasks.

### Generation task

**Figure 2:**
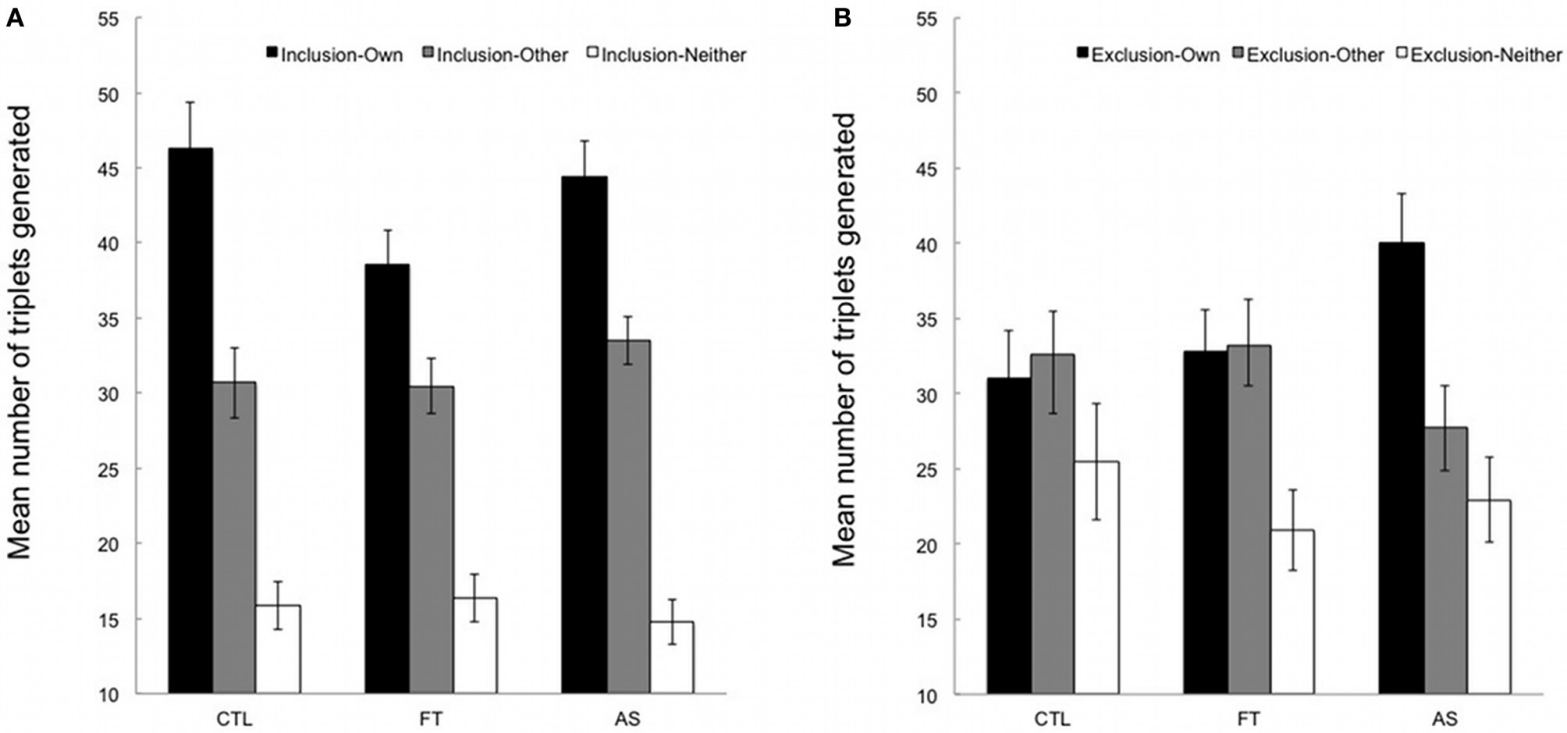
**(A)** Mean number of triplets generated in inclusion under articulatory suppression (AS), foot tapping (FT), and control (CTL) conditions. “Own”, number of SOC triplets generated from the training sequence; “other”, number of triplets from the alternate, untrained sequence; neither, number of triplets from neither the training nor the untrained sequence. Error bars represent standard errors. **(B)** Mean number of triplets generated in exclusion under articulatory suppression (AS), foot tapping (FT), and control (CTL) conditions. “Own”, number of SOC triplets generated from the training sequence; “other”, number of triplets from the alternate, untrained sequence; neither, number of triplets from neither the training nor the untrained sequence. Error bars represent standard errors.

Figures 2A,B show the mean number of “own”, “other,” and “neither” triplets generated under inclusion and exclusion instructions, respectively. “Neither” triplets will not be considered since the focus of interest lies in the comparison between “own” and “other” triplets.

We first compared the number of “own” triplets (from the training sequence) generated under inclusion and exclusion instructions in the three conditions. An ANOVA with instructions (inclusion vs. exclusion) as a within-subjects variable and conditions (articulatory suppression, foot tapping, and control) as a between-subjects variable revealed a significant instruction effect, [*F*_(1, 53)_ = 11.67, *p* = 0.001, η^2^_*p*_ = 0.18], indicating that overall participants generated more “own” triplets in inclusion than in exclusion (*M* = 43.05, *SD* = 11.58 and *M* = 33.55, *SD* = 13.39, respectively). The main effect of condition was marginally significant, [*F*_(2, 53)_ = 3.08, *p* = 0.054, η^2^_*p*_ = 0.10], suggesting that the overall number of triplets generated varied across conditions (*M* = 38.66, *SD* = 6.41 in the control condition, *M* = 35.72, *SD* = 8.23 in the foot tapping condition, and *M* = 42.22, *SD* = 9.15 in the articulatory suppression condition). The instruction × condition interaction failed to reach significance, [*F*_(2, 53)_ = 1.93, *p* = 0.16, η^2^_*p*_ = 0.07]. However, the pattern of results indicates that more “own” triplets were generated under exclusion instructions in the articulatory suppression group only. Considering that we had a strong *a priori* hypothesis regarding the effect of articulatory suppression on exclusion performance, and that the *F*-value of the interaction was above 1 (see Wilcox, 1987 for details), we carried out planned contrasts on the mean number of “own” triplets generated under inclusion and exclusion instructions separately. As expected, inclusion performance in the articulatory suppression group did not differ from the other groups, *t*(53) = 0.61, *p* > 0.50. In contrast, and crucially for the purpose of the present study, participants in the articulatory suppression condition generated significantly more “own” triplets in exclusion than participants in the control and foot tapping conditions (*M* = 40.01, *SD* = 3.20 for articulatory suppression condition vs. *M* = 31.95, *SD* = 2.05 for control and foot tapping conditions taken together), *t*(53) = 2.17, *p* < 0.05.

Additional paired-samples *t*-tests compared the number of “own” and “other” triplets generated under inclusion and exclusion instructions for each of the three groups, hence providing an appropriate chance level^1^. Participants generated significantly more “own” than “other” triplets under inclusion instructions in all groups, *t*(18) = 2.95, *p* < 0.01, *t*(18) = 2.97, *p* < 0.01, and *t*(17) = 2.92, *p* = 0.01 in the control, foot tapping, and articulatory suppression, respectively. Thus, participants demonstrated above-baseline sequence knowledge in the inclusion task, irrespective of whether they had to perform a concurrent secondary task or not, and irrespective of the nature of the secondary task. A different pattern of results emerges under exclusion instructions. While a similar number of “own” and “other” triplet was generated both in the control and foot tapping conditions, all *t*s < |1|, *p*s > 0.70, participants generated significantly more “own” than “other” triplets (*M* = 40.01, *SD* = 3.20 vs. *M* = 27.72, *SD* = 2.77) in the articulatory suppression condition, *t*(17) = 2.33, *p* < 0.05. This suggests that participants were specifically unable to withhold their responses during the exclusion task when an articulatory suppression task had to be performed concurrently.

Taken together, our data suggest that the acquired sequential knowledge was available in a direct generation test. Above-chance inclusion scores in all conditions indicate that participants were able to recruit this knowledge when necessary. In exclusion, participants demonstrated some level of control over the expression of their knowledge, not only when they performed the task alone in the control condition, but also when a concurrent foot-tapping task was added. Conversely, and as predicted, a detrimental effect of articulatory suppression was observed, above and beyond the costs associated with the requirement of performing two tasks at the same time.

### Recognition task

Participants were required to react to sequences of three elements (triplets) by pressing the key corresponding to the location of the stimulus (as in the SRT task) and to rate from 1 to 6 the extent to which they felt these sequences were old or new (i.e., “own” vs. “other”). Sequences with erroneous responses were excluded. Mean recognition ratings for both types of sequence (“own” vs. “other”) are plotted separately for the three conditions. High ratings correspond to judgments of novelty and are expected for “other” triplets, whereas low ratings correspond to judgements of oldness and are expected for “own” triplets.

**Figure 3:**
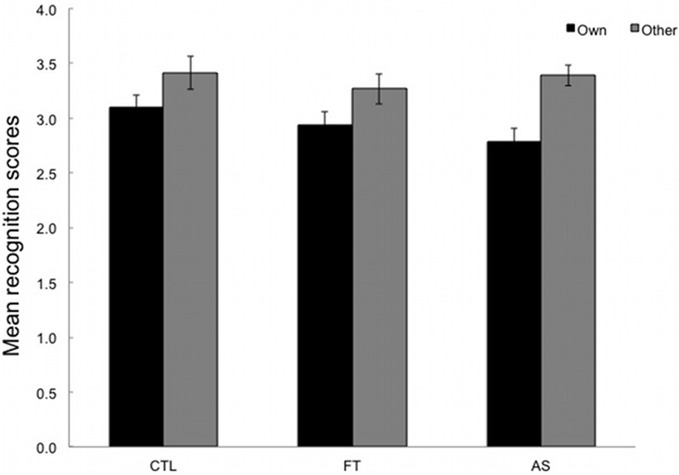
**Mean recognition ratings given for the 24 test triplets, plotted separately for the three conditions: articulatory suppression, foot tapping, and control.** Low ratings (between 1 and 3) are expected for old (“own”) triplets whereas high ratings (between 4 and 6) are expected for new (“other”) triplets. Error bars represent standard errors.

As can be seen in Figure 3, “own” and “other” triplets are overall correctly discriminated. An ANOVA with type of sequence (“own” vs. “other”) as within-subjects variable and condition (articulatory suppression, foot tapping, and control) as between-subjects variables yielded a significant main effect of type only, [*F*_(1, 53)_ = 26.36, *p* < 0.001, η^2^_*p*_ = 0.33]. Neither the main effect of condition, nor the type × condition interaction reached significance, *F* < 1 and [*F*_(2, 53)_ = 1.23, *p* = 0.28, η^2^_*p*_ = 0.05, respectively]. Overall this indicates that participants were able to differentiate between “own” and “other” triplets (*M* = 2.95, *SD* = 0.51 and *M* = 3.36, *SD* = 0.57). More importantly, this ability to recognize parts of the training sequence did not differ across conditions.

## Discussion

Consider the familiar “Neither yes nor no” game, in which one is repeatedly asked “Yes/No” questions under the constraint that his/her answers should be neither “yes” nor “no”. Winning the game requires tight executive control as one has to continuously refrain from the strong, prepotent tendency to respond to the questions in the familiar manner, that is, by saying either “yes” or “no”. Inevitably, as attention wanes, one comes to the point where one answers “without thinking about it” by producing precisely the answer that had to be avoided. Should we therefore consider this automatic, uncontrolled responding as inherently unconscious? We do not think so. Most people indeed realize that they failed the game immediately after having uttered the taboo words “yes” or “no” or even while uttering them: they blush, chuckle, say “oops,” and so on. This indicates that they were perfectly aware of the inadequacy of their response, but just could not help it.

The “neither yes nor no” game is a good illustration of the phenomenon we sought to explore with this study. Participants were first incidentally trained to become sensitive to sequential regularities in a SRT task. In a subsequent generation task, they were then asked to generate these sequential regularities under inclusion instructions, that is, under conditions where both automatic and controlled processes contribute to increasing performance. Next, participants had to carry out the same generation task, but this time under exclusion instructions, that is, under instructions to specifically avoid producing the learned sequential regularities. In other words, the prepotent tendency to reproduce what they had been trained on now had to be suppressed, just as a “yes” or “no” response has to be withheld in the game. Under such exclusion instructions, automatic and controlled processes thus act in opposition, for expressing automatic knowledge cannot be refrained. In the final recognition task participants had to discriminate between fragments that were part of the training sequence or not. They were required to express their knowledge of the sequence through a continuous scale, with build-in confidence judgements.

Thus, the generation task under exclusion instructions and the recognition task can be conceived as reflecting different aspects of our ability to use knowledge: Exclusion task performance provides an index of the extent to which such knowledge can be controlled, while recognition task performance provides and index of the extent to which this knowledge is consciously accessible (see Gaillard et al., 2009 for a similar reasoning in aging, but see Rünger and Frensch, 2010 for a critique of the use of direct tests to measure consciousness). Any dissociation between performance in the exclusion subtask and in the recognition task would therefore demonstrate the absence of a systematic association between control and consciousness. Such a dissociation is exactly what we observed when participants performed the generation task without the possibility to rely on inner speech to help inhibiting sequential fragments in the exclusion subtask.

Blocking inner speech thus increased impulsive responding as in the Go/No-Go task (Tullett and Inzlicht, 2010). That is, participants in the articulatory suppression group were not able to withhold the tendency to reproduce the training sequence under exclusion instructions, whereas participants in the control and foot tapping groups demonstrated that ability. One might argue that those between-group differences may be due to pre-existing differences in a number of basic cognitive abilities, such as fluid intelligence or working memory. However, such differences have little bearing on implicit learning (Feldman et al., 1995; Unsworth and Engle, 2005; Kaufman et al., 2010). The influence of processing speed is less clear. On the one hand, Kaufman and colleagues (2010) observed a link between processing speed and implicit learning but they reckon that the nature of this link remains unclear. On the other hand, small to non-existent correlations have been shown between incidental learning measures and processing speed (Feldman et al., 1995). Thus we are rather confident that the specific difficulties observed in the exclusion performance with concurrent articulation suppression do not stem from any baseline group differences. Moreover and most crucially, this impaired performance cannot be explained either by insufficient knowledge of the sequence or by lack of conscious knowledge of the sequence. Indeed, participants in the articulatory suppression group did not differ from the other groups in the incidental learning phase. Under inclusion instructions, they also retrieved the appropriate sequential knowledge and produced sequential (“own”) fragments similarly to control and foot tapping participants. In addition, they were able to make conscious (correct) decisions about the extent to which sequential fragments were familiar or not, just as other participants, in the recognition task. It is important to keep in mind that the recognition scores in our experiment did not only consist in judging whether a given triplet was part of the training sequence, as in Yes/No binary responses, but also required participants to indicate how confident they were in their judgment. Thus our data suggest that conscious access and executive control might not be always associated after all. This conclusion is very much in line with Tzelgov's (1997) theory of automaticity, in which automaticity is characterized not by the fact that it involves unconscious knowledge, but rather by the fact that behavior guided by automatic knowledge has a ballistic character, that is, that once initiated, it unfolds of its own until the learned effect is obtained. Thus, according to Tzelgov, we are (at least potentially) aware of most automatic behavior—it is just that such behavior can no longer be the object of executive control.

Our results are also consistent with Cleeremans' theory of automaticity (Cleeremans, 2008), according to which both executive control and availability to consciousness depend on representation quality, where quality involves graded dimensions such as strength, stability in time, and distinctiveness, all driven by learning mechanisms the computational objective of which is to increase overall adaptation. Weak representations, typical of implicit cognition (e.g., subliminal perception) are of poor quality and hence are only weakly available to conscious awareness. Such representations also do not require conscious control for they only exert weak effects on behavior. Very strong representations, on the other hand, are characteristic of automaticity, and likewise do not require cognitive control in virtue of the fact that they are adapted. The strength of such representations makes them available to conscious awareness in a manner that the weak representations characteristic of implicit cognition cannot achieve. Thus, with automatic behavior, consciousness has become optional: The knowledge is available to conscious inspection, but such conscious monitoring is not necessary for the knowledge to drive behavior in an adapted manner. Note, however, that such adaptation can fail. For instance, it is almost impossible for a continental pedestrian not to turn his head leftwards when crossing a street in London, for the learned behavior is so automatic that it can only be prevented from being triggered by environmental cues with considerable effort. Likewise, the ironic instruction “Do not think of a white bear” (Wegner et al., 1987) is almost impossible to follow. It should be clear that these examples of maladaptive automated behavior are the exception rather than the rule, however. It is also striking to note that in all such cases, one is acutely aware of the existence of a conflict and of our inability to overcome it.

Congruently, our data suggests that knowledge that cannot be controlled is available to awareness. It is interesting to reflect upon other possible patterns of dissociation between executive control and conscious awareness. In particular, one may wonder whether full control is possible in the absence of awareness. Intuitively, this should be possible, since after all, it is precisely what happens when we are behaving automatically. This pattern of dissociation is what Dienes and Perner (2007) described as involving “COLD control”, that is, as involving executive control without higher-order thought (HOT, Rosenthal, 1997). In the cold control theory of hypnosis, Dienes and Perner argue that hypnosis offers promising ways to study executive control without conscious awareness. For instance, they report an experiment in which participants counted six fingers on their hand, after they received the hypnotic suggestion to forget the number “4”. Overriding the tendency to count “1, 2, 3, 4, 5” fingers on a hand requires executive control and yet, participants deny awareness of why they counted six fingers on their hand.

What distinguishes our approach of the consciousness-control problem from the cold control perspective is that our participants were all fully aware during the experiment, (i.e., they were not in any modified state of consciousness). Moreover, all our stimuli were supraliminal, as opposed to studies exploring the relationship between consciousness and control with subliminal stimuli (Heinemann et al., 2009; van Gaal et al., 2009; Kiefer and Martens, 2010; Capa et al., 2011). However, some of them could not recruit inner speech as self-cuing aid during the generation task. This resulted in a specific detrimental effect of articulatory suppression, (i.e., concurrent foot tapping did not result in less control over the sequence under exclusion instructions). This is reminiscent of previous data obtained with the task-switching paradigm (Emerson and Miyake, 2003; Miyake et al., 2004; Saeki and Saito, 2004, 2009; Bryck and Mayr, 2005; Saeki et al., 2006). The involvement of inner speech in cognitive control is further suggested by the relationship between inner speech production and increased activity in the left inferior frontal gyrus (LIFG), which is similarly recruited during working memory tasks (see Morin, 2009).

Thus our results clearly suggest that inner speech plays an important role in the recall and control of implicitly acquired sequence knowledge. To the best of our knowledge, only one study investigated the role of inner speech in sequence learning (Farley et al., 2007, Exp. 3–4). However, Farley and colleagues intended to disrupt sequence learning itself (and not the product of learning, i.e., sequential representations) with irrelevant speech, on the ground that irrelevant speech is detrimental to serial order processing. Irrelevant speech lengthened reaction times in the SRT task, but did not prevent learning of the sequence. This is further evidence that the role inner speech plays is a specific one. Blocking inner speech is not detrimental to automatic tasks such as sequence learning but it impairs performance in tasks requiring endogenous control, as the generation-exclusion subtask.

### Conflict of interest statement

The authors declare that the research was conducted in the absence of any commercial or financial relationships that could be construed as a potential conflict of interest.

## Footnotes

^1^The appropriate baseline level of performance in the generation task consists in the comparison between “own” and “other” triplets. Indeed, the alternative “other” sequence is structurally identical to the training “own” sequence, except that the former was trained and the latter was not (Wilkinson and Shanks, 2004).
